# The Protective Impact of Black Chokeberry Fruit Extract (*Aronia melanocarpa* L.) on the Oxidoreductive System of the Parotid Gland of Rats Exposed to Cadmium

**DOI:** 10.1155/2019/3403264

**Published:** 2019-11-23

**Authors:** Zofia Dąbrowska, Ewa Dąbrowska, Barbara Onopiuk, Paweł Onopiuk, Karolina Orywal, Barbara Mroczko, Małgorzata Pietruska

**Affiliations:** ^1^Medical University of Białystok, M. Skłodowskiej-Curie 24 A, Białystok 15-276, Poland; ^2^Department of Gerostomatology of Medical University of Białystok, ul. Akademicka 3, Białystok 15-286, Poland; ^3^Medical University of Białystok and Private Dental Office in Białystok, ul. Rzemieślnicza 37, Białystok 15-773, Poland; ^4^Otholaryngology Department, Medical University of Białystok, M. Skłodowskiej-Curie 24 A, Białystok 15-276, Poland; ^5^Department of Biochemical Diagnostics, Medical University of Białystok, ul. Waszyngtona 15A, Białystok 15-269, Poland; ^6^Department of Periodontology, Medical University of Białystok, ul. Waszyngtona 13, Białystok 15-269, Poland

## Abstract

Cadmium (Cd) is a strongly toxic heavy metal with prooxidative properties. Since the exposure of the general population to this metal is predicted to increase, effective methods are being sought to prevent its negative actions. One of them involves the use of the antioxidant potential of polyphenol compounds contained in black chokeberry fruit extract and their capability of complex formation with Cd^2+^. The study objective was to investigate whether the administration of A. melanocarpa fruit extract rich in polyphenol compounds during low and moderate exposures to cadmium can protect the parotid gland against oxidative damage. The study was conducted using the experimental model on female Wistar rats which were given 0.1% aqueous extract of *Aronia melanocarpa* fruit (AE) and/or cadmium at a concentration of 1 (Cd_1_) or 5 (Cd_5_) mg Cd/kg feed for 3 and 10 months, and on control animals. The exposure to Cd attenuated the enzymatic antioxidant barrier (catalase (CAT), superoxide dismutase (SOD), glutathione peroxidase (GPx)) and increased the concentration of hydrogen peroxide (H_2_O_2_), protein carbonyl (PC) groups, and oxidized lipids (LPO) in parotid gland. These disorders led to a reduction in the total antioxidative status (TAS), an increase in the total oxidative state (TOS), and development of stress. The administration of AE at both levels of exposure to cadmium substantially improved the enzymatic antioxidant barrier (CAT, SOD, GPx) and prevented oxidative damage to cellular macromolecules (PC, LPO) and the increase in the level of H_2_O_2_, MPO, TOS, and stress indicator (OSI = TOS/TAS) in the parotid gland. Concluding, it should be stated that the consumption of aronia products may prevent oxidative/antioxidative imbalance induced by Cd and oxidative stress development in the parotid gland, thus protecting the gland from damage.

## 1. Introduction

Cadmium (Cd) occupies the top place in the group of toxic metals and its harmful effects in living organisms have been relatively well known. The results reported in recent years have provided evidence that even low chronic exposure to this metal is hazardous to health [1–4]. The effects of exposure to cadmium involve damage to numerous tissues and organs, including the kidneys [5, 6], liver [7, 8], bone system [9, 10], cardiovascular system [11, 12], and contribute to the development of neoplastic diseases [13, 14] and oral disorders [15–17]. Smoking is the primary source of Cd exposure, especially in relation to oral health, mainly in people living in the areas with low level of cadmium pollution and no occupational exposure. The effect of the Cd in tobacco on oral mouth is gingival pigmentation, darkening teeth, slow regeneration of the soft tissues, and dysphagia [18, 19].

The ability of Cd to induce oxidative stress is one of the main toxic mechanisms affecting the body. The mechanism of stress development in conditions of exposure to cadmium is multidirectional [20–23]. The Cd^2+^ ions do not exhibit the ability to directly generate reactive oxygen species (ROS), but they may cause oxidative damage to numerous tissues and organs, including the salivary glands via intermediate mechanisms (attenuation of the protective antioxidant barrier, reduced thiol status, and release of transitory metal ions) [20, 22, 24–30].

The effect of Cd on salivary gland tissue has not been studied so extensively as its other effects and it is thus not fully elucidated. The available findings, however, indicate that the exposure to this metal may lead to functional lesions and structural damage to the salivary glands, what was shown in authors' previous research [31–33]. Moreover, it may be the consequence of, e.g., its prooxidative effect [27, 28].

Due to high toxicity of cadmium, its wide spread in the environment, and the prognosis that its exposure among the general population is going to increase [1, 2], a search has been conducted for the effective methods to prevent health effects of the exposure, especially its deleterious effect on the parotid gland tissue. Natural substances with their antioxidant properties should be considered, including polyphenol compounds commonly encountered in the products of plant origin, to effectively prevent the impact of the prooxidative influence of cadmium (Cd) exposure [22, 26, 34–38]. The antioxidant properties of polyphenol compounds result to a large extent from the presence of hydroxyl groups (-OH) in their molecules, which are able to form metal ion complexes, e.g., of cadmium, and to inactivate free radicals and reactive oxygen species [37, 39].

Black chokeberry fruits are among the richest sources of these compounds [22, 35]. The available data indicate that the administration of A. melanocarpa extract decreases cadmium accumulation in the body and protects from damage to the bone structure and liver [21, 26, 29, 30]. Therefore, and regarding its high antioxidant potential [35–37], the extract may also be assumed to protect against the development of oxidative stress in the parotid gland.

## 2. Aim

In the current study, we aimed to determine the possible protective effect of A. melanocarpa fruit extract on the rat parotid gland in conditions of low and moderate exposures to cadmium.

## 3. Materials and Methods

### 3.1. Black Chokeberry Fruit Extract

Extract of AMP was received from Adamed Consumer Healthcare (Tuszyn, Poland). According to the producer, the extract contained 65.74% of polyphenols, including 18.65% of anthocyanins (Certificate KJ 4/2010; Butch No. M100703). The total polyphenol content in the powdered AMP extract assayed by us (according to the colorimetric Folin-Ciocalteu method described by Tawaha et al.) [40] reached 61.24 ± 0.01% (mean ± SD).

Extracts of AMP compounds are anthocyanins, proanthocyanidins (oligomeric and polymeric catechins), flavonols (glycoside derivatives of quercetin), and hydroxycinnamic acids (chlorogenic and neochlorogenic acids). The anthocyanin profile of Aronia melanocarpa consists almost exclusively of cyanidin glycosides, namely, cyanidin-3-arabinoside, cyanidin-3-galactoside, cyanidin-3-glucoside, and cyanidin-3-xyloside (the first two are predominant), and relatively low amounts of pelargonidin-3-arabinoside and pelargonidin-3-galactoside. Flavonols were identified as the minor class of polyphenols in chokeberries. Quercetin, kaempferol, and several quercetin mono- and diglycosides (quercetin-3-galactoside, quercetin-3-glucoside, quercetin-3-rutinoside, quercetin 3-vicianoside, and quercetin-3-robinobioside) were also detected in aronia berries, but in relatively low concentrations. Chokeberry proanthocyanidins consist exclusively of (-)-epicatechin units bonded by a C4 to C8 linkage. The hydroxycinnamic acids are represented by significant amounts of chlorogenic and neochlorogenic acids [41, 42].

### 3.2. Animals and Experimental Protocol

The parotid salivary glands of the rats used in the current study were collected and secured during an experiment conducted in the Department of Toxicology, Medical University of Białystok. The study was approved by the Local Ethics Committee for Experiments on Animals in Białystok, nr 60/2009.

The experiment was conducted on 96 young (3-4-week-old) female Wistar [Crl: WI (Han)] rats with an initial body weight of approximately 50 g. The animals were kept in stainless steel cages in standard breeding conditions (relative humidity 50 ± 10%, temperature 22 ± 2°C, 12-hour cycle) throughout the experiment. The rats were divided randomly into 6 experimental groups.

The animals were randomized into 6 study groups of 16:

Group 1—control: throughout the experiment (3 or 10 months), the rats received purified water and standard fodder (Labofeed, Kcynia)

Group 2—AMPF: the rats received only a 0.1% water solution of extract of polyphenols (AMPF) for drinking for 3 or 10 months and standard fodder

Group 3—Cd^1^: the rats were exposed to cadmium (as CdCl_2_) in the fodder, receiving 1 mg Cd/kg for 3 or 10 months, and received purified water for drinking

Group 4—Cd_1_ + AMPF: throughout the period (3 or 10 months) of exposure to 1 mg of Cd/kg, the rats received the 0.1% water solution of extract of polyphenols from black chokeberry fruit

Group 5—Cd^5^: the rats received fodder containing 5 mg Cd/kg for 3 or 10 months and purified water for drinking

Group 6—Cd_5_ + AMPF: during exposure (for 3 or 10 months) to 5 mg Cd/kg of fodder, the rats received 0.1% water solution of extract of polyphenols from black chokeberry fruit for drinking

The powdered black chokeberry fruit extract was obtained from Adamed Consumer Healthcare (Tuszyn, Poland), which according to the producer contained 65.74% of polyphenol compounds, including 18.65% of anthocyanins. Polyphenolic compounds and cadmium were administered orally. By administering a 0.1% polyphenol's extract water solution from black chokeberry fruit, it was ensured that the rats would consume no less than 51.7-104.6 mg/kg of body weight, depending on the age of the animal.

AMP were administered in the form of a 0.1% aqueous extract of these compounds from the aronia berries administered as the only drinking fluid. This fluid was prepared daily by dissolving the appropriate amount of the powdered AMP extract in appropriate volume of redistilled water (1 g of the extract per litter of redistilled water). Polyphenol concentration, determined by us (as reported by Tawaha et al. [40]), in such prepared formulation was stable during 24 h and reached 0.612 ± 0.006 mg/mL (mean ± SD). The last group of rats, that drank water without AMP addition and was feed with the standard Labofeed diets without Cd (Labofeed H and B diets), served as a control. Redistilled water (containing <0.05 *μ*g Cd/L and not completely deprived of necessary bioelements) was used as drinking water to eliminate Cd intake in the control group and AMP group, and additional Cd consumption in the animals exposed to this metal. Cd concentration in the standard Labofeed H and B diets (without Cd addition) did not differ and reached 0.0584 ± 0.0049 mg/kg (mean ± SD), whereas this metal concentration in the 0.1% water extract of AMP was <0.05 *μ*g/L. The experimental model used allowed to estimate the impact of AMP on the body turnover of Cd at very low (control group vs. AMP group), low (Cd^1^ group vs. Cd^1+^ AMP group), and moderate (Cd^5^ group vs. Cd^5+^ AMP group) chronic exposure to Cd. Exposed rats to 1 mg Cd/kg of fodder are equivalent to low environmental exposure of humans to this metal, while administering 5 mg Cd/kg of fodder is equivalent to moderate exposure of humans.

During the whole experiment, the animals were housed under controlled conventional conditions (temperature 22 ± 2°C, relative humidity 50 ± 10%, 12/12 h light-dark cycle). Animals had free access to drinking water and food during the whole experiment. In the last week of the 3^rd^, 10^th^ month of the experiment 8 rats of each group, and all survived animals (8 rats of the control, Cd^1+^ AMP, and Cd^5+^ AMP groups, and 7 rats of the AMP, Cd^1^, and Cd^5^ groups) in the last week of the 24^th^ month were placed in metabolic cages for 24-hour urine and faeces collection for five consecutive days. During this time, the animals had free access to food and drinking water (with or without Cd and AMP depending on the experimental group); consumption of which was monitored. The urine and faeces were removed from the metabolic cages every 24 h. The urine was centrifuged (MPW-350R centrifugator, Medical Instruments, Warsaw, Poland) immediately after collection and its volume was measured. The 24-hour urine and faeces collected during the 5-day period were pooled for further analysis.

After the finish treatment, the animals were deprived of food overnight and next they were subjected to barbiturate anaesthesia (Morbital, 30 mg/kg b.wt, i.p.). The salivary parotid glands were rinsed thoroughly in ice-cold 0.9% NaCl (physiological saline). Next, they were weighed with an automatic balance (OHAUS®. Nanikon, Switzerland; accuracy to 0.0001 gg). The biological material not used immediately was stored frozen at -80°C until assayed.

The experimental model was described in detail by Brzóska et al. [21].

### 3.3. Analytical Procedures

The parotid glands were homogenized using a knife homogenizer (Ultra-Turrax T25, IKA) in cold phosphate buffer (50 mM, pH = 7.4) to obtain 10% homogenates. Each homogenate was divided into two parts—one portion was centrifuged (MPW-350R, Medical Instruments) 700 × g for 20 minutes (to determine catalase (CAT), myeloperoxidase (MPO), total antioxidant status (TAS), total oxidant status (TOS), glutathione (GSH), hydrogen peroxide (H_2_O_2_), lipid peroxidation (LPO), and protein carbonyl groups (PC)). The other portion was centrifuged 20,000 × g for 30 minutes (to determine glutathione peroxidase (GPx) and superoxide dismutase (SOD)) at 4°C [43].

TAS and TOS were determined using diagnostic kits ImAnOx (TAS) Kit and PerOx (TOS) Kit, Immundiagnostik AG. The TAS values determined in control samples (CTRL1—211.5 ± 16.1 *μ*mol/dm^3^ and CTRL2—246 ± 8.485 *μ*mol/dm^3^) confirmed accurateness of the measurements. The precision of the method expressed as coefficient of variation (CV) was <5%. The TOS values measured in control samples (CTRL1—193.3 ± 13.1 *μ*mol/dm^3^ and CTRL2—441.6 ± 18.45 *μ*mol/dm^3^) were within the ranges provided by the producer. The method precision expressed as coefficient of variation (CV) was <5.4%. The stress index (OSI) was mathematically calculated as the TOS/TAS ratio.

The activity of CAT was determined by the spectrophometric method according to Aebi [44]. The method precision expressed as coefficient of variation (CV) was <6%.

The activity of GPx (BIOXYTECH GPx-340™ Assay), level of H_2_O_2_ (BIOXYTECH H_2_O_2_-560™ Assay), and level of LPO (BIOXYTECH LPO-586™ Assay) were determined using diagnostic kits of Percipio Biosciences. The method precision expressed as coefficient of variation (CV) was <2.2%, <4.5%, and <5%, respectively.

The activity of SOD (Superoxide Dismutase Assay Kit) and the level of GSH (Glutathione Assay Kit) were measured using diagnostic kits of Cayman. The method precision expressed as coefficient of variation (CV) was <2.5% and <4.5%, respectively.

The activity of MPO (Rat MPO ELISA Kit) was determined by a diagnostic kit of SunRed. The method precision expressed as coefficient of variation (CV) was <5%.

The level of PC was determined by the spectrophotometric method according to Reznick and Packer [45]. The method precision expressed as coefficient of variation (CV) was <5.6%.

In order to express the parameters on protein basis, total protein concentration was assessed using a diagnostic kit of BioMaxima, where method precision was expressed as coefficient of variation (CV < 4.8%).

The level of cadmium in the salivary glands was determined using the atomic absorption spectroscopy (AAS) in graphite cuvette. The level of Cd measured in the simultaneously analyzed reference material was consistent with the value provided by the producer (Bovine muscle, ERM-BB184, Belgium). The method precision expressed as coefficient of variation (CV) was <2%.

All the abovementioned tests were conducted according to the producers' instructions. The measurements were performed using the spectrometer UV VIS SPECORD 50 PLUS (Analytik Jena, Jena, Germany), Epoch microplate reader (BioTek Instruments, Inc.; Winooski, USA), and an automatic washer for Wellwash 4 microplates (Thermo LabSystems, Helsinki, Finland). The level of cadmium was determined using the atomic absorption spectrophotometer (HITACHI Z-5000, Japonia) equipped with a graphite cuvette (Pyro cuvette A, Hitachi) and a hollow cathode lamp for metal determination with the AAS method (Photron, Narre Warren, Australia).

### 3.4. Statistical Analysis

Statistical analysis of the study results was conducted using the computer program Statistica 13 (StatSoft; Tulsa, USA). The results were presented as mean ± SD for 8 rats in each of the experimental groups. In order to assess the statistical variability of differences between the experimental groups, a nonparametric Kruskal-Wallis test was performed. The independent and interactive impact of cadmium and the extract of polyphenol compounds derived from black chokeberries on the indicators of the oxidoreductive status were evaluated using the two-way analysis of variance (ANOVA/MANOVA). The analysis of Spearman's coefficient of correlation was also carried out between Cd and the indicators of the oxidoreductive status. Differences between the groups were considered statistically significant at *p* < 0.05.

## 4. Results

### 4.1. Indicators of the Antioxidant Status in Parotid Gland Tissue

The values of the indicators of the antioxidant status of the rat parotid gland are presented in Table 1. The administration of AE to the study animals for 3 and 10 months had no impact on the activities of CAT, SOD, or GPx and the levels of GSH and TAS in the salivary gland examined. The use of 1 and 5 mg Cd/kg of feed for 3 months reduced the activity of CAT compared to the control and did not affect the activity of SOD and GPx and the level of TAS. Prolongation of exposure to 10 months (1 and 5 mg Cd/kg feed) resulted in reduced GPx and SOD activity. TAS concentration was only lower at the 1 mg Cd/kg dose. No changes were found in the activity of CAT in comparison with the control. Moreover, after the two periods (3 and 10 months) and levels (1 and 5 mg Cd/kg feed) of Cd exposure, no changes were observed in the level of the nonenzymatic antioxidant GSH compared to the control group.

The administration of AE during exposure to 1 mg Cd/kg feed for 3 months led to an increase in the activities of CAT and SOD as compared to the group receiving only Cd^1^. In the Cd^5+^ AE group of animals, an increase in TAS concentration was found relative to the control group. In addition, GPx activity was higher than in the group receiving Cd^5^ only. The prolongation of the exposure to 10 months in the Cd^1+^ AE group resulted in an increase in the activity of CAT as compared to the control rats and receiving only Cd^1^. However, the activities of SOD and GPx were higher in rats exposed to Cd^1^ alone. The administration of AE in the case of higher level of exposure to cadmium (Cd^5+^ AE) increased the activities of CAT and GPx concentration as well as TAS as compared to control animals and those exposed only to 5 mg Cd/kg feed. It also caused a rise in the activity of SOD as compared to the rats receiving 5 mg Cd/kg feed only. In addition, administration of AE rats for 3 and 10 months when exposed to 1 and 5 mg Cd/kg feed did not affect GSH concentration.

The ANOVA/MANOVA analysis (Table 2) suggests that the improvement in the antioxidant status of the submandibular gland of rats receiving AE during exposure to cadmium (1 and 5 mg Cd/kg feed) was due to the extract action and interactions of its components with Cd.

### 4.2. Indicators of the Oxidant Status in Parotid Gland Tissue

The values of the indicators of the oxidant status of the rat parotid gland are presented in Table 3. The administration of AE to the animals for 3 and 10 months had no effect on the levels of MPO, H_2_O_2_, LPO, PC, TOS, and stress indicator OSI. In rats exposed to cadmium (1 and 5 mg Cd/kg feed) for 3 months, the concentration of PC and H_2_O_2_ increased, while the concentration of TOS and LPO increased at a higher level of exposure (5 mg Cd/kg of feed), as compared to control animals. Prolongation of the exposure to 10 months resulted in an increase in the levels of PC, TOS, and OSI at the exposure to 1 and 5 mg Cd/kg feed as compared to control animals. With the higher exposure, a rise was also observed in the levels of H_2_O_2_ and LPO in comparison with control. The application of AE during exposure to 1 mg Cd/kg feed for 3 months caused a decrease in TOS, OSI, H_2_O_2_, and PC as compared to group of animals receiving only Cd^1^. When the exposure was higher (Cd^5+^ AE), the levels of LPO and TOS as well as OSI were lower than in the group of animals exposed to Cd^5^ alone and did not differ from the control. In addition, administration of AE to animals during exposure to 1 mg Cd/kg feed did not affect MPO and LPO levels. The prolongation of the exposure to 10 months resulted in a decrease in OSI, MPO, and PC in the group receiving Cd^1+^ AE as compared to the levels observed in rats exposed to Cd^1^ only and did not differ from the control. With the higher exposure to cadmium (Cd^5+^ AE), a decrease was noted in TOS, OSI, and PC as compared to the group given Cd^5^ alone and did not differ from control rats. In addition, administration of AE to animals during exposure to 5 mg Cd/kg feed did not affect the concentration of MPO, LPO, and H_2_O_2_ in the salivary gland tested.

The ANOVA/MANOVA analysis (Table 4) indicates that the reduction in the oxidant status of the parotid gland of rats receiving AE during exposure to cadmium (1 and 5 mg Cd/kg feed) was due both to the independent effect of the extract and the interactions of its components with Cd. However, the independent effect of AE seems to be stronger.

### 4.3. Cadmium Concentration

Cd levels in the rat parotid gland are presented in Table 5.

In the rats exposed to 1 and 5 mg Cd/kg feed, for 3 and 10 months, the level of this metal in the parotid gland was higher than in the control. The administration of AE during cadmium exposure (Cd^1+^ AE and Cd^5+^ AE) resulted in reduced accumulation of this metal in the gland as compared to the animals receiving Cd^1^ and Cd^5^ alone. However, the levels of this metal in these groups (Cd^1+^ AE and Cd^5+^ AE) were higher than in the control group. As shown by the ANOVA/MANOVA variance analysis (Table 6), the level of cadmium in the parotid gland of rats receiving *Aronia melanocarpa* extract depended on the independent cadmium action.

Numerous correlations were noted between the level of Cd and the indicators of the oxidoreductive status of the parotid gland studied (Table 7). All the indicators of the oxidation state (TOS, OSI, MPO, PC, H_2_O_2_, LPO) were positively correlated with cadmium exposure. A negative correlation was observed between OSI and the activity of antioxidant enzymes (CAT, SOD, GPx) and TAS. A positive correlation was found between OSI and indicators of the oxidant state (MPO, PC, LPO, H_2_O_2_) and TOS.

## 5. Discussion

Oxidative stress exerts an unfavorable effect on the human body and is a major factor in the pathomechanism of many systemic diseases. Its involvement in the etiology of the diseases affecting the circulatory system, nervous system, osseous system, and kidney [20, 24, 25, 46, 47], as well as in the pathogenesis of oral disorders [48–53] has been described.

Experimental studies conducted in recent years allowed researchers to state that oxidative stress is also a mechanism underlying the toxic action of cadmium. The mechanism of stress development in conditions of exposure to cadmium is multidirectional [20, 22–25]. Cd^2+^ ions are not capable to directly generate reactive oxygen species (ROS), but they can lead to, via indirect mechanisms, oxidative injuries in numerous tissues and organs, including the salivary glands. These mechanisms involve disorders in the mitochondrial respiratory chain, attenuation of the protective antioxidant barrier, reduction in the thiol status, and release of transitory metal ions [22, 23].

Increased generation of reactive oxygen species and oxidative damage to macromolecules and cell organelles are due to cadmium-induced attenuation of cell antioxidant potential. ROS, as highly reactive structures, easily come into interactions with proteins, sugars, polyunsaturated fatty acids, and nucleic acids, leading to disorders in their biological functions [20, 22, 24, 25].

The exposure of rats to 1 mg Cd/kg feed corresponds to low environmental human exposure to this metal, whereas a dose of 5 mg Cd/kg feed reflects moderate exposure. The levels of Cd in blood and urine of animals exposed to 1 and 5 mg Cd/kg feed were in the range of 0.1030–0.3060 *μ*g/dm^3^ and 0.0852–0.2762 *μ*g/g creatinine and 0.5840–1.3320 *μ*g/dm^3^ and 0.2839–0.8197*μ*g/g creatinine [21]. The same ranges of Cd levels can be observed in the residents of highly industrialized countries, which may indicate that the experimental model applied well reflects the environmental exposure of the general population to this metal. The ranges of Cd levels noted in rats exposed to 5 mg Cd/kg feed or even much higher are observed in heavy smokers.

We found that cadmium leads to the development of oxidative stress in the parotid gland by damaging the enzymatic antioxidant barrier. We observed that the administration of 1 and 5 mg Cd/kg feed to rats for 3 months caused a reduction in the activity of CAT by 66% and 52%, respectively. No changes were noted in the activity of SOD, GPx, and the level of TAS. The prolongation of the exposure to 10 months (1 and 5 mg Cd/kg feed) resulted in a decrease in the activity of SOD by 72% and 56% and GPx by 40% and 32%. TAS concentration was only lower in rats receiving 1 mg Cd/kg feed by 33%. No changes, however, were found in the activity of CAT, what may be induced by organism adaptation to long Cd exposure. Moreover, after both periods (3 and 10 months) and levels (1 and 5 mg Cd/kg feed) of exposure to cadmium, no changes were revealed in the level of the nonenzymatic antioxidant GSH.

In the current study, in rats exposed to cadmium in both concentrations (1 and 5 mg Cd/kg feed), the level of PC was increased by 51% and 68%, respectively, and H_2_O_2_ by 36% and 31%, respectively, and TOS and LPO at the higher level of exposure (5 mg Cd/kg feed) by 79% and 2.1-fold, respectively, as compared to control animals after 3 months of exposure. The prolongation of the exposure to 10 months resulted in an increase in the levels of PC, TOS, and OSI by 97%, 2- and 3.2-fold, respectively, at the exposure to 1 mg Cd/kg feed and 2.3-fold, 2.8-fold, and 3.5-fold, respectively, at the exposure to 5 mg Cd/kg feed as compared to control animals. At the higher level of the exposure, an increase was also noted in the level of H_2_O_2_ (by 64%) and LPO (by 63%) in comparison with the control group.

The oxidative damage observed in our research to proteins and lipids with a simultaneous increase in H_2_O_2_ and the inhibition of the activity of antioxidant enzymes in rat parotid gland tissue seem to indicate the development of oxidative stress in this gland. This reasoning is confirmed by the finding of oxidative/antioxidative imbalance in the tissue examined, which was reflected in the increased TOS/TAS ratio being a stress marker (OSI) in the parotid gland already after the 3-month exposure to 5 mg Cd/kg feed and after the 10-month exposure to 1 and 5 mg Cd/kg feed.

Importantly, we found that the consumption of black chokeberry fruit extract during exposure to cadmium can protect, at least partly, against disorders in the enzymatic and nonenzymatic antioxidant barrier and prevent oxidative damage to cell macromolecules in parotid gland tissue.

The administration of AE to animals during exposure to 1 mg Cd/kg feed for 3 months increased the activity of CAT (2.4-fold) and SOD (by 66%) as compared to the group receiving Cd^1^ only. In the Cd^5+^ AE group, an increase was found in the activity of TAS (by 33%) compared to the control group. Moreover, the activity of GPx was higher (2.1-fold) than in the Cd^5^ group. The prolongation of the exposure to 10 months in the Cd^1+^ AE group resulted in the increased activity of CAT in comparison with control rats and those receiving Cd^1^ only, 2.1-fold and 2.5-fold, respectively. However, the activities of SOD and GPx concentration were higher than in the rat group exposed to Cd^1^ only 2.8- and 2.2-fold, respectively. The administration of AE at the higher level of exposure to cadmium (Cd^5+^ AE) caused an increase in the activity of CAT (by 63% and 2-fold, respectively), GPx (by 94% and 2.8-fold), and TAS (by 85% and 2.2-fold, respectively) as compared to control animals and rats exposed only to 5 mg Cd/kg feed. It also brought an increase in the activity of SOD in comparison with rats receiving only 5 mg Cd/kg feed by 92%. Importantly, the administration of *A. melanocarpa* fruit extract during exposure to 5 mg Cd/kg feed already after 3 months completely prevented cadmium-induced reduction in the activity of GPx and even increased TAS as compared to the control. However, the administration of AE during exposure to 1 or 5 mg Cd/kg feed for 10 months fully protected the activities of CAT, SOD i GPx, and TAS concentration against reduction and even at a higher level of exposure increased the activities of CAT, GPx, and TAS concentration compared to the control. As shown by the analysis of variance ANOVA/MANOVA, the beneficial effect of the extract administration was associated with its independent action and with interactions of its components with Cd. Taking into account the results of this analysis and the fact that *A. melanocarpa* fruit extract is characterized by strong antioxidant properties [35, 37, 54–60], and that its administration during cadmium exposure increased the antioxidant potential of the parotid gland, it can be assumed that the beneficial effect of the extract on the activity of the enzymes and the levels of GSH and TAS was due to its antioxidant effect.

The administration of AE to animals during exposure to 1 mg Cd/kg feed for 3 months resulted in lower values of TOS (by 59%), OSI (by 65%), H_2_O_2_ (by 31%), and PC (by 49%) than in the group of rats receiving Cd^1^ only. At the higher level of exposure (Cd^5+^ AE), the concentrations of LPO, TOS, and OSI values were lower than in the group of animals exposed only to Cd^5^ by 47%, 59%, and 63%, respectively, and did not differ from the control group. The prolongation of the exposure to 10 months caused a reduction in OSI (by 55%), MPO (by 34%), and PC (by 49%) in the Cd^1+^ AE group as compared to rats exposed to the metal alone (Cd^1^) and did not differ from the control. At the higher level of exposure to cadmium (Cd^5+^ AE), TOS, OSI, and PC were reduced by 39%, 73%, and 24%, respectively, in comparison with the animals receiving only Cd^5^. An important finding was that the administration of *A. melanocarpa* fruit extract during exposure for 3 months to cadmium can protect against the increase in the levels of TOS and OSI at both levels of exposure to this metal, H_2_O_2_ and PC at 1 mg Cd/mg feed, and LPO at 5 mg Cd/mg feed. In turn, the 10-month administration of AE in animals exposed to 1 and 5 mg Cd/mg feed completely prevented the Cd-induced increase in OSI and PC, MPO concentration at 1 mg Cd/mg feed, and in TOS at 5 mg Cd/mg feed. The two-way analysis of variance ANOVA/MANOVA showed that the beneficial effect of *A. melanocarpa* fruit extract on the indicators of the oxidative status of the parotid gland is due to an independent action of the extract as well as interactions of its components with cadmium. Importantly, the relatively high content of polyphenol compounds in black chokeberry fruit [35–37, 56, 57] and their ability to chelate toxic heavy metal ions [39] allow the assumption that these compounds play a major role in the prevention of oxidative damage to cell macromolecules (PC, LPO) and of an increase in the levels of H_2_O_2_, MPO, TOS, and OSI in the parotid gland.

In the light of the available data, the independent protective impact of black chokeberry fruit extract on the enzymatic and nonenzymatic antioxidant barrier, indicators of the oxidant status, and the degree of damage to oxidant cell macromolecules in the parotid gland can be explained by high antioxidant potential of this extract determined mainly by polyphenol compounds [35, 37, 56, 57]. Our earlier research conducted using the same experimental model as the one applied in the current study revealed that the administration of black chokeberry fruit extract to rats chronically exposed to cadmium enhances the enzymatic and nonenzymatic antioxidant barrier, protects from excessive accumulation of H_2_O_2_, reduces the level of PC, LPO, and MPO, and decreases TOS and OSI in bone tissue [26] and liver [29, 30].

The interactive effect of A. *melanocarpa* fruit extract and cadmium on the oxidoreductive status in the parotid gland can be justified by the ability of the extract component, especially polyphenol compounds [34, 39, 59], as well as fiber, tannins, and pectin [35, 57, 58] to bind divalent metal ions. The presence of a large number of hydroxyl groups in the molecule enables these compounds to form complexes with metal ions [35–37, 56, 57] and facilitates chelation of toxic metal ions, including cadmium, as well as metals that catalyze oxidation reactions, such as copper(I) and iron(II) [39, 60]. It is thus assumed that the polyphenol compounds present in A. *melanocarpa* fruit can be responsible for Cd^2+^ uptake in the lumen of the alimentary tract and in this way reduce its absorption and bioavailability [21, 59]. We found that both the low and moderate exposures to cadmium from 3 to 10 months increased its level in the gland examined, whereas administration of 0.1% extract of A. *melanocarpa* fruit during exposure to this xenobiotic decreased its accumulation in the parotid gland. This allows the suggestion that the beneficial effect of the extract consumption was, at least partly, caused by diminished absorption and accumulation of cadmium in the body and in consequence prevented negative health effects caused by this toxic metal, including its prooxidant action. Moreover, numerous correlations between cadmium and indicators of the oxidant/antioxidant status in animals simultaneously receiving A. *melanocarpa* extract and cadmium in the diet seem to confirm the contribution of the antioxidant properties of the extract in the mechanism of its protective impact on the parotid gland.

The current report is one of the first to assess the effect of *A. melanocarpa* fruit extract on the oxidoreductive balance in the parotid gland in conditions of low and moderate exposures to Cd. However, some experimental studies have proved that such polyphenol compounds as curcumin and resveratrol show a protective action against oxidative stress, but not cadmium-induced, in the salivary glands [60, 61].

In summary, it should be stated that even low exposure to cadmium may lead to enzymatic disorders (reduced activity of CAT, SOD, GPx) of the antioxidant barrier, cause oxidative disorders of important cell macromolecules (increased PC), reduce total antioxidant status TAS, and increase total oxidant state TOS and stress indicator OSI. The consumption of black chokeberry fruit extract during low and moderate exposures to this xenobiotic increases the activity of CAT, SOD, GPx, and TAS and prevents the Cd-induced increase in the levels of PC, LPO, MPO, H_2_O_2_, TOS, and OSI in the parotid gland. Our results seem to confirm the hypothesis that the consumption of black chokeberry products during low and moderate exposures to cadmium in the diet may have a protective effect on the parotid gland and prevent its damage.

## 6. Conclusions

Our study results contribute to the current knowledge of the protective role of *Aronia melanocarpa* fruit extract on the parotid gland during exposure to cadmium and confirm the benefits of black chokeberry fruit preserves in the prevention of unfavorable effects of this heavy metal on the oral health.

## Figures and Tables

**Table 1:** Indicators of the antioxidant status of the parotid gland in rats.

	TAS nmol/mg protein	CAT mmol H_2_O_2_/min/mg protein	SOD U/mg protein	GPx mU/mg protein	GSH nmol/mg protein
3 months					
Control	222.4 ± 18.89	20.45 ± 2.173	0.526 ± 0.048	124.2 ± 13.40	3.160 ± 0.497
AE	218.0 ± 13.64	19.46 ± 2.063	0.480 ± 0.043	122.0 ± 11.12	5.088 ± 0.920
Cd^1^	208.9 ± 15.36	6.980 ± 0.426^a,‡,b,‡^	0.406 ± 0.029	75.17 ± 9.086	4.226 ± 0.710
Cd_1_ + AE	258.2 ± 19.24	16.48 ± 1.104^c,†^	0.676 ± 0.059^c,†^	105.4 ± 8.003	4.405 ± 0.516
Cd^5^	254.9 ± 10.77	9.790 ± 0.980^a,^^∗^^,b,^^∗^	0.556 ± 0.068	88.90 ± 6.468	2.858 ± 0.821
Cd_5_ + AE	296.4 ± 28.30^a,^^∗^^,b,^^∗^^,c,^^∗^	14.05 ± 1.130^a,†^	0.602 ± 0.049	186.6 ± 9.204^c,‡,d,^^∗^^,e,‡^	5.477 ± 0.655

10 months					
Control	184.8 ± 7.274	14.68 ± 1.301	0.583 ± 0.070	80.96 ± 5.248	3.311 ± 0.750
AE	185.1 ± 26.85	14.61 ± 1.415	0.512 ± 0.114	100.4 ± 15.53	2.853 ± 0.648
Cd^1^	124.0 ± 9.215^a,^^∗^	12.07 ± 0.854	0.161 ± 0.020^a,‡,b,^^∗^	48.20 ± 5.031^a,^^∗^	2.822 ± 0.400
Cd_1_ + AE	200.5 ± 15.38^c,^^∗^	30.76 ± 3.087^a,^^∗^^,b,^^∗^^,c,‡^	0.449 ± 0.062^c,†^	108.0 ± 11.43^c,†^	3.242 ± 0.462
Cd^5^	155.1 ± 7.972	11.93 ± 1.087^d,‡^	0.255 ± 0.025^a,^^∗^	55.32 ± 5.433^a,^^∗^^,d,^^∗^	2.338 ± 0.330
Cd_5_ + AE	341.5 ± 18.63^a,^^∗^^,b,^^∗^^,c,‡,e,†^	23.92 ± 2.447^a,^^∗^^,c,‡,e,^^∗^	0.545 ± 0.076^c,‡,e,^^∗^	157.3 ± 21.90^a,^^∗^^,b,‡,c,‡,e,‡^	5.346 ± 1.506^b^

Data is mean ± SD for 8 rats. Statistically significant differences (nonparametric Kruskal-Wallis test). ^a^Compared to control. ^b^Compared to AE group. ^c^Compared to Cd^1^ group. ^d^Compared to Cd^1+^ AE group. ^e^Compared to Cd^5^ group; ^∗^*p* < 0.05, ^†^*p* < 0.01, ^‡^*p* < 0.001, respectively.

**Table 2:** Estimation of the main and interactive effects of Cd and AE on the indicators of the antioxidant status of the parotid gland in rats.

	TAS	CAT	SOD	GPx
Main effect of Cd	3.906^a^	NS	NS	NS
Main effect of AE	9.741^†^	15.12^‡^	NS	23.09^‡^
Interactive effect of Cd and AE	10.67^†^	18.30^‡^	11.33^†^	14.30^‡^

Data present *F* values and level of statistical significance. ^†^*p* < 0.01, ^‡^*p* < 0.001, ^a^*p* = 0.05, NS *p* > 0.05.

**Table 3:** Indicators of the oxidant status of the parotid gland in rats.

	TOS (nmol/mg protein)	OSI (TOS/TAS)	MPO (ng/mg protein)	H_2_O_2_ (nmol/mg protein)	LPO (nmol/mg protein)	PC (nmol/mg protein)
3 months						
Control	24.66 ± 3.716	0.112 ± 0.016	6.184 ± 0.500	6.743 ± 0.765	1.862 ± 0.167	26.98 ± 2.99
AE	22.11 ± 2.446	0.104 ± 0.014	4.915 ± 0.447	6.416 ± 0.614	1.916 ± 0.204	19.98 ± 1.455
Cd^1^	29.98 ± 4.387	0.145 ± 0.021	7.317 ± 0.665	9.204 ± 0.642^a,^^∗^	2.235 ± 0.270	40.85 ± 2.279^a,^^∗^^,b,†^
Cd_1_ + AE	12.22 ± 1.642^c,^^∗^	0.051 ± 0.009^c,†^	5.136 ± 0.515	6.371 ± 0.666^c,^^∗^	1.771 ± 0.144	20.83 ± 2.357^c,^^∗^
Cd^5^	44.16 ± 8.377^a,^^∗^^,b,^^∗^^,d,†^	0.173 ± 0.032^d,†^	7.444 ± 0.476	8.835 ± 0.304^a,^^∗^	3.927 ± 0.915^a,^^∗^^,b,^^∗^^,d,^^∗^	45.40 ± 6.907^a,^^∗^^,b,‡,d,†^
Cd_5_ + AE	18.18 ± 4.155^e,†^	0.064 ± 0.016^c,^^∗^^,e,^^∗^	5.803 ± 0.482	7.446 ± 0.473	2.098 ± 0.208^e,^^∗^	28.93 ± 1.719

10 months						
Control	24.63 ± 8.303	0.132 ± 0.040	5.828 ± 0.586	6.427 ± 0.332	1.800 ± 0.306	19.60 ± 1.660
AE	20.03 ± 3.535	0.122 ± 0.025	3.898 ± 0.372	5.946 ± 0.481	1.772 ± 0.253	19.38 ± 3.165
Cd^1^	48.37 ± 8.682^a,^^∗^^,b,^^∗^	0.419 ± 0.095^a,^^∗^^,b,^^∗^	7.045 ± 0.668	7.334 ± 0.557	2.153 ± 0.217	38.57 ± 6.440^a,^^∗^
Cd_1_ + AE	36.13 ± 6.036	0.188 ± 0.032^c,^^∗^	3.840 ± 0.265^c,^^∗^	7.232 ± 0.475	1.664 ± 0.125	19.68 ± 3.169^c,^^∗^
Cd^5^	69.46 ± 10.11^a,†,b,‡^	0.464 ± 0.077^a,†,b,^^∗^	9.192 ± 1.407	10.52 ± 1.446^a,^^∗^^,b,†^	2.942 ± 0.285^a,^^∗^^,d,^^∗^	45.05 ± 6.289^a,†,b,†,d,†^
Cd_5_ + AE	42.29 ± 3.989^b,†,e,^^∗^	0.125 ± 0.011^e,^^∗^	6.496 ± 0.494	8.837 ± 0.678^b,†^	2.144 ± 0.190	34.08 ± 4.822^e,^^∗^

Data is mean ± SD for 8 rats. Statistically significant differences (nonparametric Kruskal-Wallis test). ^a^Compared to control. ^b^Compared to AE group. ^c^Compared to Cd^1^ group. ^d^Compared to Cd^1+^ AE group. ^e^Compared to Cd^5^ group; ^∗^*p* < 0.05, ^†^*p* < 0.01, ^‡^*p* < 0.001, respectively.

**Table 4:** Estimation of the main and interactive effects of Cd and AE on the indicators of the oxidation state of the parotid gland in rats.

	TOS	OSI	MPO	H_2_O_2_	LPO	PC
Main effect of Cd	11.85^‡^	7.539^†^	10.51^†^	17.16^‡^	5.793^∗^	26.77^‡^
Main effect of AE	8.091^†^	10.46^†^	24.19^‡^	4.700^∗^	4.017^∗^	19.95^‡^
Interactive effect of Cd and AE	4.043^†^	8.656^†^	NS	NS	4.260^∗^	7.002^†^

Data present *F* values and the level of statistical significance. ^∗^*p* < 0.05, ^†^*p* < 0.01, ^‡^*p* < 0.001, NS *p* > 0.05.

**Table 5:** The levels of cadmium in the parotid gland.

	3-month exposure	10-month exposure
Cd *μ*g/g tissue		
Control	0.0344 ± 0.0099	0.0950 ± 0.0024
AE	0.0075 ± 0.0007	0.0939 ± 0.0020
Cd^1^	0.4053 ± 0.0294^a,^^∗^^,b,^^∗^	0.7328 ± 0.0417^a,^^∗^^,b,^^∗^
Cd_1_ + AE	0.2442 ± 0.0300^a,^^∗^^,c,^^∗^	0.5876 ± 0.0318^a^^∗^^c^^∗^
Cd^5^	1.4871 ± 0.0294^a,‡,b,‡,d,^^∗^	2.0430 ± 0.0913^a,‡,b,‡,d,^^∗^
Cd_5_ + AE	1.0370 ± 0.0982^a,†,b,‡,e,^^∗^	1.7370 ± 0.0578^a,‡,b,‡,e,^^∗^

Data is mean ± SD for 8 rats. Statistically significant differences (nonparametric Kruskal-Wallis test). ^a^Compared to control. ^b^Compared to AE group. ^c^Compared to Cd^1^ group. ^d^Compared to Cd^1+^ AE group. ^e^Compared to Cd^5^ group; ^∗^*p* < 0.05, ^†^*p* < 0.01, ^‡^*p* < 0.001, respectively.

**Table 6:** Estimation of the main and interactive effects of Cd and AE on the parotid gland.

	Main effect of Cd	Main effect of AE	Interactive effect of Cd and AE
Cd	**76.00** ^‡^	**NS**	**NS**

Data present *F* values and level of statistical significance. ^‡^*p* < 0.001, NS *p* > 0.05.

**Table 7:** Analysis of the correlation between the study parameters of the oxidoreductive status of the parotid gland and cadmium concentration.

	Cd	TOS	TAS	OSI	CAT	SOD	GPx	GSH	MPO	PC	H_2_O_2_
TOS	0.482^‡^										
TAS	NS	NS									
OSI	0.356^‡^	0.908^‡^	-0.496^‡^								
CAT	NS	NS	0.290^‡^	-0.212^∗^							
SOD	-0.216^∗^	-0.395^‡^	0.614^‡^	-0.585^‡^	0.277^†^						
GPx	NS	-0.215^∗^	0.610^‡^	-0.418^‡^	0.467^‡^	0.499^‡^					
GSH	NS	NS	0.346^‡^	NS	0.210^∗^	0.204^∗^	0.392^‡^				
MPO	0.371^‡^	0.348^‡^	NS	0.250^∗^	-0.378^‡^	NS	-0.263^†^	NS			
PC	0.575^‡^	0.445^‡^	NS	0.305^†^	-0.285^†^	NS	NS	NS	0.504^‡^		
H_2_O_2_	0.534^‡^	0.396^‡^	0.273^†^	0.259^∗^	NS	NS	NS	NS	0.325^†^	0.766^‡^	
LPO	0.390^‡^	0.356^‡^	0.249^∗^	0.214^∗^	NS	NS	NS	NS	0.349^‡^	0.742^‡^	0.665^‡^

^∗^ *p* < 0.05, ^†^*p* < 0.01, ^‡^*p* < 0.001, NS *p* > 0.05.

## Data Availability

This is the Data Availability statement for the manuscript titled “The Protective Impact of Black Chokeberry Fruit Extract (*Aronia melanocarpa* L.) on the Oxidoreductive System of the Parotid Gland of Rats Exposed to Cadmium”. The data of the materials and methods and conclusions to support the findings of this study are included within the article. If any other data may be needed, please contact the corresponding author upon request.
